# Modeling spatiotemporal abundance and movement dynamics using an integrated spatial capture–recapture movement model

**DOI:** 10.1002/ecy.3772

**Published:** 2022-07-15

**Authors:** Nathan J. Hostetter, Eric V. Regehr, Ryan R. Wilson, J. Andrew Royle, Sarah J. Converse

**Affiliations:** ^1^ Washington Cooperative Fish and Wildlife Research Unit, School of Aquatic and Fishery Sciences University of Washington Seattle Washington USA; ^2^ Applied Physics Laboratory Polar Science Center, University of Washington Seattle Washington USA; ^3^ Marine Mammals Management United States Fish and Wildlife Service Anchorage Alaska USA; ^4^ United States Geological Survey Eastern Ecological Science Center Laurel Maryland USA; ^5^ United States Geological Survey, Washington Cooperative Fish and Wildlife Research Unit, School of Environmental and Forest Sciences and School of Aquatic and Fishery Sciences University of Washington Seattle Washington USA; ^6^ Present address: United States Geological Survey, North Carolina Cooperative Fish and Wildlife Research Unit, Department of Applied Ecology North Carolina State University Raleigh North Carolina USA

**Keywords:** abundance, data integration, movement, polar bear, search–encounter, spatial capture–recapture, telemetry

## Abstract

Animal movement is a fundamental ecological process affecting the survival and reproduction of individuals, the structure of populations, and the dynamics of communities. Methods to quantify animal movement and spatiotemporal abundances, however, are generally separate and therefore omit linkages between individual‐level and population‐level processes. We describe an integrated spatial capture–recapture (SCR) movement model to jointly estimate (1) the number and distribution of individuals in a defined spatial region and (2) movement of those individuals through time. We applied our model to a study of polar bears (*Ursus maritimus*) in a 28,125 km^2^ survey area of the eastern Chukchi Sea, USA in 2015 that incorporated capture–recapture and telemetry data. In simulation studies, the model provided unbiased estimates of movement, abundance, and detection parameters using a bivariate normal random walk and correlated random walk movement process. Our case study provided detailed evidence of directional movement persistence for both male and female bears, where individuals regularly traversed areas larger than the survey area during the 36‐day study period. Scaling from individual‐ to population‐level inferences, we found that densities varied from <0.75 bears/625 km^2^ grid cell/day in nearshore cells to 1.6–2.5 bears/grid cell/day for cells surrounded by sea ice. Daily abundance estimates ranged from 53 to 69 bears, with no trend across days. The cumulative number of unique bears that used the survey area increased through time due to movements into and out of the area, resulting in an estimated 171 individuals using the survey area during the study (95% credible interval 124–250). Abundance estimates were similar to a previous multiyear integrated population model using capture–recapture and telemetry data (2008–2016; Regehr et al., Scientific Reports 8:16780, 2018). Overall, the SCR–movement model successfully quantified both individual‐ and population‐level space use, including the effects of landscape characteristics on movement, abundance, and detection, while linking the movement and abundance processes to directly estimate density within a prescribed spatial region and temporal period. Integrated SCR–movement models provide a generalizable approach to incorporate greater movement realism into population dynamics and link movement to emergent properties including spatiotemporal densities and abundances.

## INTRODUCTION

Animal movement is a fundamental ecological process (Nathan et al., 2008; Turchin, 1998). Movement is driven by individual (e.g., sex, age) and spatiotemporal (e.g., habitat) factors that subsequently affect demographic rates, population structure, and community dynamics (Kays et al., 2015; Morales et al., 2010). Movement processes are of inherent interest (Hooten et al., 2017; Morales et al., 2004), whereas identifying the influence of movement on population‐level processes such as resource selection, spatiotemporal abundances, and population dynamics is necessary for a deeper understanding of how individuals and populations respond to their environments (Hays et al., 2016; Kays et al., 2015; Morales et al., 2010). Although there have been recent advancements in movement modeling (e.g., Hooten et al., 2017) and spatial population ecology (e.g., Royle et al., 2013), a unified framework for modeling movement and population dynamics is lacking (McClintock et al., 2021; Morales et al., 2010).

Spatial capture–recapture (SCR) is a dynamic set of methods used to study abundance, density, and demography of animal populations (Royle et al., 2013). SCR is based on a thinned point process model, which extends to multiple sampling methods and ecological investigations (e.g., Bischof et al., 2020; Glennie et al., 2019; Linden et al., 2018; Royle et al., 2017; Sutherland et al., 2015). To date, most SCR models have assumed that individuals maintain static home ranges within a season, where individual space use is modeled as a monotonic decline with distance from an “activity center” (e.g., Efford, 2019; Royle et al., 2013). Although some SCR extensions relax the assumption of bivariate normal space use (e.g., Linden et al., 2018; Murphy et al., 2016; Royle et al., 2016; Sutherland et al., 2015), few have explicitly modeled realistic movement processes (please refer to review in McClintock et al., 2021). Extending SCR models to include movement processes (i.e., integrated SCR–movement models; McClintock et al., 2021) provides new opportunities to connect movement dynamics to population‐level processes.

The widespread use of telemetry data allows increasingly complex investigations into movement and behavioral ecology (Hooten et al., 2017; Kays et al., 2015). Connecting telemetry data to population‐level patterns is key to understanding movement ecology and how movement affects spatiotemporal abundances and population dynamics (Hays et al., 2016; Morales et al., 2010; Nathan et al., 2008). Integrating telemetry and SCR data within an SCR–movement model is a natural link to connect telemetry data to population‐level processes (McClintock et al., 2021). Although SCR–movement models can be fit with SCR data alone, the integration of telemetry data will often be essential for the inclusion of realistic movement processes (Gardner et al., 2022) and can extend telemetry data to population‐level inferences on movement, resource selection, and distribution.

Here, we present an integrated SCR–movement model that jointly describes the distribution of individuals across a landscape, how individuals move, and how movement processes affect exposure to sampling. We conducted a simulation study to evaluate model performance and then applied the model to a study of polar bears (*Ursus maritimus*) in the eastern Chukchi Sea using a combination of SCR and telemetry data. Polar bears are distributed across the circumpolar Arctic and are listed as threatened under the United States Endangered Species Act (USFWS, 2008). Capture–recapture methods are commonly used to estimate polar bear abundance and density (e.g., Bromaghin et al., 2015; Lunn et al., 2016; Regehr et al., 2018), whereas telemetry studies are applied to investigate bear movements and their association with landscape features (e.g., Laidre et al., 2013; Wilson et al., 2014). On the spring sea ice, bears do not display a traditional activity center. Instead, individuals make directional movements in search of foraging or mating opportunities (Laidre et al., 2013), often covering areas larger than the extent of capture–recapture surveys (Bromaghin et al., 2015, Lunn et al., 2016, Regehr et al., 2018). As such, non‐spatial approaches to estimating density cannot disentangle the movement and abundance processes, resulting in abundance estimates that reflect the cumulative number of individuals exposed to sampling across the survey period (i.e., superpopulation). Under these conditions, SCR–movement models provide multiple advantages, including an explicit description of population‐level movement dynamics, spatially and temporally defined abundance and densities, and the ability to integrate multiple data sources. Conversely, the lack of an explicit movement process in standard SCR models limits the integration of detailed telemetry data and the ability to jointly investigate hypotheses linking movement, population, and landscape ecology.

## METHODS

### Model overview

We first describe a general SCR–movement model for the joint estimation of movement, abundance, and detection from SCR data. We parameterize an individual‐level movement process within an SCR framework to describe how an individual's daily location changes through time (i.e., its trajectory), and the probability of detecting an individual conditional on its trajectory. The SCR–movement model can be expressed as a state space model with three components: (i) the abundance and distribution of individuals on the first sampling occasion, (ii) movement of individuals through time, and (iii) spatial encounters of individuals. Specifically,

(1) $$\left[s_{i1}|\;\theta \right]$$

represents the initial distribution of i=1,2,…,N individuals

(2) $$\left[s_{\mathrm{it}}|s_{i\left(1:t-1\right)},\theta \right]$$

represents the movement model for t=2,3,…,T occasions, and

(3) $$\left[y_{\mathrm{it}}|s_{\mathrm{it}},\theta \right]$$

the observation process where θ denotes a vector of all unknown parameters (i.e., parameters describing initial distribution, movement, and detection) and $s_{\mathrm{it}}$ denotes the daily average location of individual i on day t. The movement model informs how an individual's daily location changes through time, which extends to various movement processes (e.g., random walk, correlated random walk; Hooten et al., 2017; Morales et al., 2004). To reflect our case study, we use daily time steps (t), however, the interval can be chosen based on study design, ecological context, and computational considerations. Finally, spatial encounter histories ($y_{\mathrm{it}}$) arise from an observation model with parameters describing the detection process conditional on the trajectory of an individual.

### Case study

**FIGURE 1:**
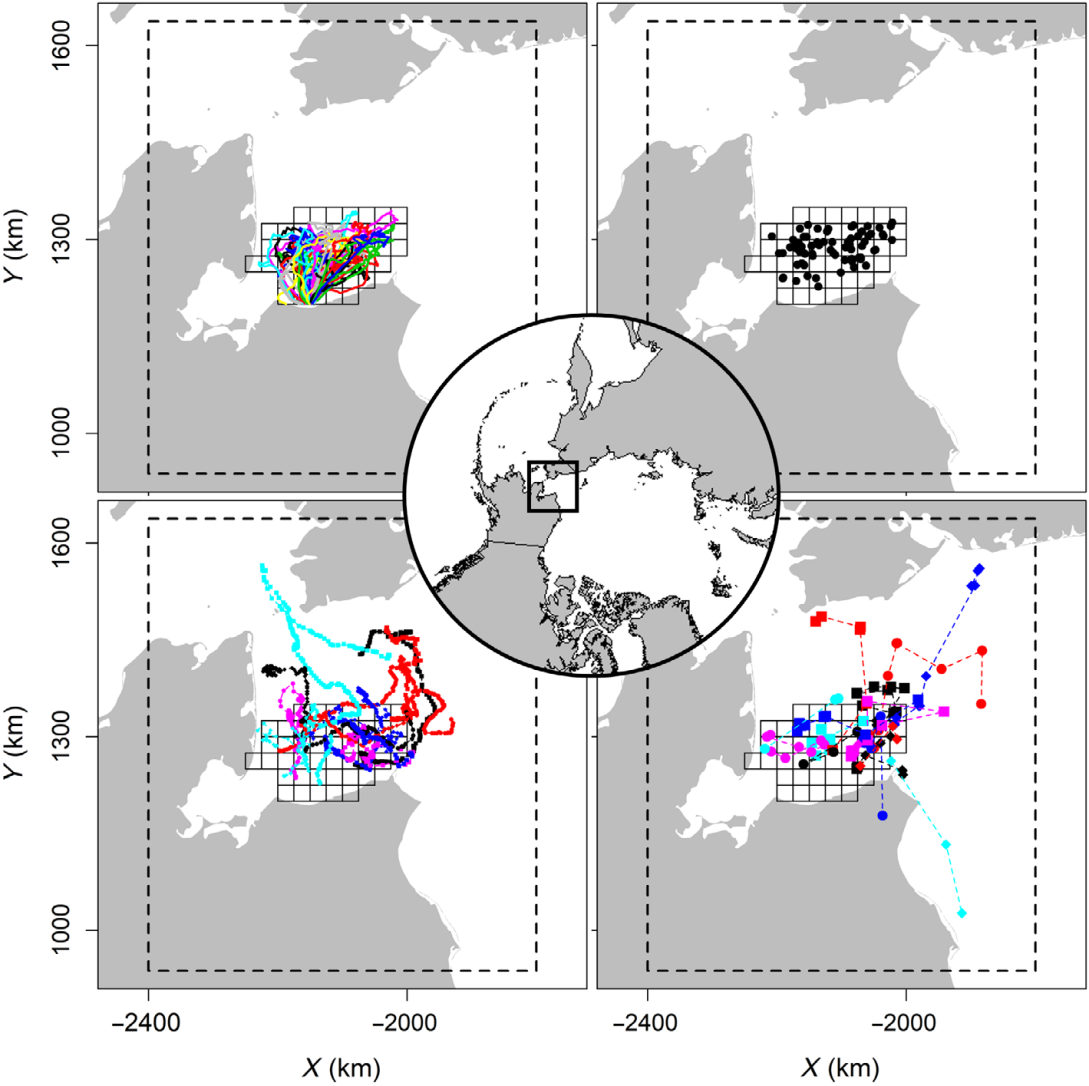
Polar bear survey and data collection during 25 March to 29 April 2015 in the eastern Chukchi Sea. Data included helicopter surveys (top left), capture–recapture event locations (top right), GPS telemetry locations (14 female bears; bottom left), and Argos telemetry locations (15 male bears; bottom right). Surveyed area (45 25 × 25 km grid cells) and state space (dashed polygon) are also shown. Colors and symbols denote different days for helicopter surveys and unique bears for GPS and Argos telemetry panels.

From 25 March to 29 April 2015, researchers conducted helicopter surveys for polar bears in the Chukchi Sea west of Alaska, USA (28 surveys spanning 36 days; Figure 1). During each survey, observed bears were captured using standard chemical immobilization techniques (Stirling et al., 1989) and painted with a unique temporary mark to allow for within‐season resights (Figure 1). Researchers also recorded the sex of each bear and, for adult females with dependent offspring, the number of cubs (ages 0–2 years old). GPS collars were applied to 15 females (ages 6–24 years old) and Argos satellite telemetry system tags (from this point forwards Argos tags) were applied to 14 males (ages 2–23 years old). GPS collars recorded location data every 2–4 h, whereas Argos tags recorded locations every 1–8 days (Figure 1). Additional details on capture, processing, and telemetry methods are available in Regehr et al. (2018) and Rode et al. (2015).

#### Modeling approach

Analysis of the case study follows the general approach described in the *Model Overview* with extensions to investigate (i) sex‐specific movement, (ii) directional persistence, (iii) latent group size (i.e., females with dependent offspring), and (iv) daily and cumulative bear abundance in a spatially defined region within the study area. Objective (iv) is particularly valuable for highly mobile species of conservation concern (e.g., marine mammals, carnivores), as management decisions are affected by the number of individuals within a defined spatial and temporal domain (i.e., density) as well as the cumulative number of individuals that use an area during a defined period (Hays et al., 2016; Lunn et al., 2016; Regehr et al., 2018). It is this objective that cannot be solved using either conventional movement modeling or SCR frameworks in isolation. We describe our SCR–movement model using notation from McClintock et al. (2021) with an emphasis on habitat‐influenced movement and search–encounter sampling (Russell et al., 2012) protocols used in our case study.

#### Distribution and movement

On occasion 1, we assumed that locations ($s_{i1}$) for N individuals were distributed uniformly across areas with sea ice in a defined state space (M; Figure 1). We defined M as a 420,000 km^2^ area, which extended >150 km from the survey area and >75 km from all telemetry locations (Figure 1). For computation purposes, we discretized M into 1 × 1 km grid cells with each cell assigned a 1 if it contained sea ice during the survey period and a 0 otherwise (Cavalieri et al., 1996, Wilson et al., 2016; Appendix S1:Figure S1). We modeled the grid cell of each individual on occasion 1 ($g_{i}$) as a categorical random variable with cell probabilities $\pi_{1:G}$, where $\pi_{g}$ is uniform for cells with sea ice and zero otherwise. Specifically,

(4) $$\left.g_{i}∼\text{Categorical}(\pi_{1:G}\right).$$

Each $s_{i1}$ is then assumed to be uniformly distributed within grid cell $g_{i}$ (please refer to Data S1).

We modeled the average location of individual i at occasions t=2,3,…,T ($s_{\mathrm{it}}$) as a function of the individual's previous locations ($s_{\mathrm{it}-1}$) and an explicit movement model. For this study, we evaluated two continuous‐space random walk movement models (Morales et al., 2004) to describe how the average location of individual i on day t changes through time and is influenced by distance to sea ice. We used potential functions (Brillinger et al., 2012; Hooten et al., 2017; McClintock et al., 2021; Preisler et al., 2013) to model the influence of sea ice on polar bear movement (Wilson et al., 2014). Potential functions can be conceptualized as a hilly landscape, where movements are directed toward (or away from) certain habitat characteristics based on the slope (Hooten et al., 2017, McClintock et al., 2021). Estimating the influence of habitat covariates on movement is a primary objective of some studies, however, potential functions also provide opportunities to restrict locations to ecologically relevant areas of expected use (Brillinger, 2003; McClintock et al., 2021; Preisler et al., 2013). Potential functions also do not induce a specific home range shape, but instead allow individuals to move through the landscape as a function of movement characteristics (e.g., directional persistence, step lengths) and landscape features (Hooten et al., 2017, McClintock et al., 2021). In our context, bear locations are not fully constrained to sea ice, but instead potential functions direct movements away from areas that are farther from sea ice (Appendix S1:Figure S1; Wilson et al., 2014; please refer to “Results” section).

The first movement process model was a bivariate normal random walk influenced by distance to sea ice and sex‐specific variance terms for movement ($\sigma_{\mathrm{sex}}$);

(5) $$s_{\mathrm{it}+1}∼\text{Normal}\left(s_{\mathrm{it}}+\delta \nabla c\left(s_{\mathrm{it}}\right),\sigma_{\mathrm{sex}\left[i\right]}I\right)$$

where $\;c\left(s_{\mathrm{it}}\right)$ is the distance to sea ice covariate at an individual's current location, ∇ is the gradient operator, δ controls how bear movement responds to distance to sea ice, $\sigma_{\mathrm{sex}}$ are sex‐specific movement standard deviations in the x‐ and y‐direction, and I is a 2 × 2 identity matrix. We calculated gradients using the *ctmcmove* package (Hanks, 2018), which creates a vector field of partial derivatives pointing in the direction of the greatest rate of increase in a habitat covariate, in our case, areas with minimum distance to sea ice. Therefore, δ > 0 implies bears move toward areas with sea ice and are repelled from areas with increasing distance to sea ice. The second movement model we considered was a correlated random walk with directional persistence (Morales et al., 2004) that included sex‐specific directional persistence ($\gamma_{\mathrm{sex}}$) and movement variance parameters ($\sigma_{\mathrm{sex}}^{2}$), and the distance to sea ice potential function. Specifically,

(6) $$s_{\mathrm{it}+1}∼\text{Normal}\left(s_{\mathrm{it}}+\gamma_{\mathrm{sex}\left[i\right]}\left(s_{\mathrm{it}}-s_{\mathrm{it}-1}\right)+\delta \nabla c\left(s_{\mathrm{it}}\right),\sigma_{\mathrm{sex}\left[i\right]}I\right)$$

where $\gamma_{\mathrm{sex}}$ describes the directional persistence where 0≤γ≤1. When γ=0 the movement process reverts to a bivariate normal random walk. This parameterization of a correlated random walk focuses on the location process ($s_{\mathrm{it}}$) but shares similarities to velocity models that estimate step lengths and turning angles (Hooten et al., 2017; Jonsen et al., 2005). For our case study, we expected $\gamma_{\mathrm{sex}}>0$, as telemetry data suggested bears moved with directional persistence (Laidre et al., 2013; Figure 1). We limited exploration to these two sex‐specific movement models given the relatively low number of bears detected, limited encounter histories, and sparse male telemetry data (Figure 1). The general framework described above, however, is easily extended to investigate more complex movement process and habitat relationships in continuous and discrete space (Gardner et al., 2022; McClintock et al., 2021).

#### Observation processes

Helicopter surveys occurred across *J* = 45 grid cells in 2015 (Figure 1), using 25 × 25 km grid cells to align with the resolution of sea ice imagery (≈25 × 25 km resolution). Following precedents for SCR search–encounter models (Royle et al., 2013; Russell et al., 2012), we treated each grid cell as an effective trap. Encounter histories ($y_{\mathrm{it}}$) therefore denote the grid cell of detection for individual i on day t. Individuals that were not detected were assigned $y_{\mathrm{it}}$ = J+1. Encounter history data were modeled as categorical random variables,

(7) $$y_{\mathrm{it}}∼\text{Categorical}\left(\xi_{\mathrm{it}}\right)$$

where $\xi_{\mathrm{it}}$ is a vector of length J+1 describing the probability of detecting individual i on day t in each of the J grid cells or non‐detection (J+1). We modeled $\xi_{\mathrm{it}}$ using a multinomial logit function of survey effort in cell j on day t ($x_{\mathrm{tj}}$= km surveyed in cell j on day t), an immediate behavioral response ($B_{\mathrm{it}}$= 1 for the first survey after capture, 0 otherwise), and the Euclidean distance between an individual's average daily location and the grid cell centroid ($d_{\mathrm{itj}}$;) using a half‐normal detection function with variance parameter $\sigma_{\det}^{2}$ (Russell et al., 2012). Specifically,

(8) $$\text{mlogit}\left(\xi_{\mathrm{itj}}\right)=\alpha_{0}+\alpha_{1}\log\left(x_{\mathrm{tj}}\right)+\alpha_{2}B_{\mathrm{it}}+\left(-d_{\mathrm{itj}}^{2}/2\sigma_{\det}^{2}\right).$$

This observation model accounted for unequal survey effort across space and time and the tendency to check on bears during the survey immediately following first capture.

Telemetry tagged bears provided location data independent of helicopter surveys, including data on movements beyond the surveyed area. We modeled telemetry locations ($\mu_{\mathrm{i\tau}}$) assuming,

(9) $$\mu_{i\tau }∼\text{Normal}\left(s_{\mathrm{it}},\sigma_{\det}^{2}I\right)$$

where $\mu_{i\tau }$ is a recorded telemetry location for individual i at time $\tau \in \left(t-1,t\right)$. The variance parameter ($\sigma_{\det}^{2}$) is shared across the telemetry (Equation 9) and SCR (Equation 8) observation submodels and allows us to tease apart movements within an occasion ($\mu_{i\tau }|s_{\mathrm{it}},\sigma_{\det}^{2}$) from movements across occasions ($s_{\mathrm{it}+1}|s_{\mathrm{it}},\sigma_{\mathrm{sex}},\gamma_{\mathrm{sex}},\delta$). The number of locations per day varied by transmitter settings but was restricted to a maximum of 4 (Figure 1). We followed the methods of McClintock et al. (2015) to account for Argos telemetry error, assuming that reported locations ($u_{i\tau }$) were bivariate normal random variables with mean $\mu_{i\tau }$ and a variance–covariance matrix informed from error ellipse data provided by the Argos tags.

#### Abundance

We used data augmentation to estimate the number of independent bears (N) in the state space (Royle & Dorazio, 2012). Data augmentation introduces a data set of M individuals where M≫ N and each individual has a binary inclusion parameter $z_{i}$, where $z_{i}=1$ if the individual is part of the population and 0 otherwise. In our study, all individuals were assigned a reproductive state ($r_{i}$ = 1 [male], 2 [female without dependent offspring], or 3 [female with dependent offspring]; from this point forwards “reproductive state”), with separate augmentation values for each group. Therefore,

(10) $$z_{i}∼\text{Bernoulli}\left(\psi_{r_{i}}\right)$$

where $\psi_{r}$ is the probability an individual assigned reproductive state r is part of the population (i.e., the N individuals in the state space). Reproductive state could also be modeled as a latent categorical grouping variable (Royle & Converse, 2014), however, we found that assigning augmented individuals to a reproductive state and using state‐specific augmentation values ($M_{r}$) greatly improved mixing and convergence.

Using data augmentation, the total number of independent bears in the state space (M) is derived as $N_{M}=\sum_{i=1}^{M}z_{i}$. Our primary interest, however, was the ability of SCR–movement models to estimate abundance in a defined spatial area (A) within the larger state space, specifically where A is the area encompassing the J = 45 sampled grid cells (Figure 1). To accomplish this, we monitored an indicator variable $w_{\mathrm{it}}$ during each Markov Chain Monte Carlo (MCMC) iteration, where $w_{\mathrm{it}}=1$ if $s_{\mathrm{it}}\in A$ and 0 otherwise. We then monitored daily abundance within A as $N_{At}=\sum_{i=1}^{M}z_{i}w_{\mathrm{it}}$ and the cumulative number of individuals that used area A by occasion t as $N_{At}^{*}=\sum_{i=1}^{M}z_{i}\times \max\left(w_{i1:t}\right)$, where $\max\left(w_{i1:t}\right)$ = 1 if individual i was present in area A at least once before or on occasion t. As such, $N_{AT}^{*}$ is the cumulative number of individuals that used area A during the study period (i.e., the superpopulation; Kendall et al., 1997).

For females with dependent offspring, we modeled numbers of cubs ($n_{i}|r_{i}=3\;$) as a categorical random variable. Specifically,

(11) $$n_{i}|r_{i}=3∼\text{Categorical}\left(\omega_{1:3}\right)$$

where $\omega_{1:3}$ denotes the probability that a female with dependent offspring ($r_{i}$ = 3) has 1, 2, or 3 young, reflecting the possible litter sizes for Chukchi Sea polar bears (Regehr et al., 2018). Here, $n_{i}$ is known for all observed bears but latent (i.e., NA) and estimated for all augmented individuals in reproductive class 3. We derived the total number of dependent offspring during each MCMC iteration as $N_{\text{Cubs}}=\sum_{i=1}^{M}z_{i}I\left(r_{i}\right)n_{i}$, where the indicator variable $I\left(r_{i}\right)$ denotes if the female was in reproductive class “female with offspring,” therefore incorporating uncertainty in the abundance of females with offspring ($z_{i}I\left(r_{i}\right)$) and the unknown litter sizes of those females ($n_{i}$). Total abundance is subsequently reported as the total number of bears (i.e., independent individuals + dependent offspring) unless otherwise noted.

Together, this integrated SCR–movement approach models the latent abundance and locations of bears (both observed and unobserved) at the start of the study, and movement of those bears through time. Telemetry data provide detailed information on the locations and movement of collared bears, whereas aerial survey data denote areas that were searched, locations of individually identifiable bears, and the number of dependent cubs. As such, telemetry data directly inform parameters for initial distribution, movement, space use, and latent locations of collared individuals ($\pi ,\sigma ,\sigma_{\det}^{2},\gamma ,\delta ,s_{\mathrm{it}}$). Aerial survey data are linked to those same parameters while also providing information on the detection (α), litter size (ω), and data augmentation (i.e., abundance; $z_{i}$) parameters (Appendix S2).

### Simulation study

We conducted two simulation studies to investigate the ability of SCR–movement models to estimate parameters and derive abundances without bias. The state space, habitat covariates, and survey area followed from the case study (Figure 1). We set N = 500 individuals and K = 25 days. The initial distribution of individuals was assumed to be uniform across sea ice within the state space. In the first simulation, individuals moved via a correlated random walk (σ=15.0, γ = 0.50) and surveys occurred on 20 randomly selected occasions out of the K = 25 days. To reflect stochastic survey effort, we randomly selected 20 daily helicopter tracks out of 28 possible tracks recorded during the case study (Figure 1). Detection parameters were set at $\alpha_{0}$ = −8.0, $\alpha_{1}$ = 2.5, and $\sigma_{\det}\;$= 5.0 to reproduce observed sample sizes and reflect case study results. We set the potential gradient parameter (δ = 50) to restrict individuals to locations on or near the sea ice. In the second simulation, we investigated the bivariate normal random walk with σ= 15.0, γ = 0.00 and all other parameter settings remaining the same. In all scenarios, the first 15 captured individuals received telemetry tags that provided four locations ($\mu_{i\tau }$) per occasion.

For each simulation, we generated and analyzed 100 data sets and evaluated model performance using percent relative bias and 95% credible interval (CRI) coverage of model parameters (σ, γ, $\sigma_{\det}$, $\alpha_{0}$, $\alpha_{1}$) and derived daily and cumulative abundances in the survey area ($N_{At}$ and $N_{AT}^{*}$, respectively). Our simulations were designed to reflect common challenges in capture–recapture studies of low‐density populations of highly mobile individuals, including small numbers of captured individuals (∼35–60 bears per study; mean = 46 bears), low encounter rates (e.g., ∼50%, 25%, 15%, and <10% of bears detected on 1, 2, 3, and >3 occasions, respectively), non‐uniform survey effort, and movement of individuals through the survey area during the study. Complementary simulation studies evaluating random walk and correlated random walk models using a continuously operating trapping grid are provided in Gardner et al. (2022).

### Implementation

Models were fit using MCMC methods implemented in a Bayesian framework using NIMBLE v0.10.0 (de Valpine et al., 2017) accessed through R v4.0.0 (R Core Team 2018). For simulation studies, we ran three chains for 100,000 iterations with 25,000 iterations discarded as burn‐in and thinned to every 5th iteration to reduce file size. For the case study, we increased the number of chains to five and the number of iterations to 525,000 to increase the effective number of posterior samples. We assessed convergence using diagnostic plots and the Gelman–Rubin statistic ($\hat{R}$; Gelman et al., 2013). Results are reported as posterior medians and 2.5 and 97.5 percentiles (95% CRI) of retained posterior samples.

Vague priors were used for all parameters: independent $\text{normal}\;\left(0,\mathrm{sd}=10\right)$ for detection parameters (α), $\text{gamma}\left(0.01,0.01\right)$ for standard deviations ($\sigma_{\mathrm{sex}}$, $\sigma_{\det}$), $\text{Dirichlet}\left(1\right)$ prior for litter size probabilities ($\omega_{1:3}$), and $\text{Beta}\left(1,1\right)$ for the directional persistence parameters ($\gamma_{\mathrm{sex}}$). Augmentation values were specific to each reproductive state and set at 803 for males ($M_{1}$), 480 for females without dependent offspring ($M_{2}$), and 317 for females with dependent offspring ($M_{3}$), which guaranteed $M_{r}\gg N_{r}$. We used independent $\text{Beta}\left(1,1\right)$ priors for each inclusion probability ($\psi_{1:3}$). Based on preliminary examination, we used $\text{normal}\;\left(50,\mathrm{sd}=1\right)$ for the potential function parameter (δ), which restricts locations to areas on or near the sea ice (Data S1). The potential function parameter could not be estimated as too few bears were observed near the shoreline; however, we evaluated multiple values of δ and found that results were robust to reasonable selections (e.g., similar abundance and parameter estimates for δ = 25 or 50).

The model described above can be expressed in the BUGS language (Lunn et al., 2000); however, to improve MCMC efficiency we applied a custom NIMBLE function to update locations at occasion 1 and a custom sampler to propose and evaluate full trajectories of augmented individuals (Data S1 and S2). These customizations increased effective posterior sample sizes per unit time by more than an order of magnitude, although run times for a single simulated data set still required >5 days. R scripts are provided as Data S1 (simulation) and Data S2 (case study). Case study results are also compared with results from a traditional SCR model that excludes the movement model in Appendix S3. Although not an objective of this study, this comparison demonstrates the ability of SCR–movement models to estimate a broader suite of ecologically relevant parameters linking movement, detection, and abundance (Appendices S2 and S3).

## RESULTS

### Case study

During data collection in 2015, observers recorded 73 detections of 48 independent bears (27 males, 14 females without offspring, seven females with offspring) on 28 sampling days across the 36‐day sampling period (Figure 1). Detections included 30 bears that were observed once, 14 bears observed twice, three observed on three occasions, and one bear observed on six occasions. Of the seven females with dependent offspring, two had one‐cub litters, and five had two‐cub litters. We present results from the correlated random walk model as male and female bears displayed directional persistence ($\gamma_{\mathrm{sex}}$ > 0) and abundances were similar across SCR–movement models.

Movement between occasions by male bears ($\sigma_{\text{male}}$ = 14.9 km, 95% CRI: 11.8–19.4) was generally larger than that of female bears ($\sigma_{\text{female}}$ = 11.0, 95% CRI: 10.4–11.8; Table 1), whereas directional persistence was similar across sexes ($\gamma_{\text{male}}$ = 0.47, 95% CRI: 0.25–0.64; $\gamma_{\text{female}}$ = 0.51, 95% CRI: 0.44–0.58; Table 1). Within‐occasion movement ($\sigma_{\det}$ = 5.11, 95% CRI: 5.00–5.24) was significantly less than between‐occasion movement ($\sigma_{\text{male}},\sigma_{\text{female}}$; Table 1).

**TABLE 1:** Parameter estimates (median, 95% credible interval) for movement, detection, data augmentation, and litter size from an integrated SCR–movement model using a correlated random walk (C‐RW) or bivariate normal random walk (BVN‐RW).

Parameter	C‐RW	BVN‐RW
Between‐occasion movement		
$\;\sigma_{\text{male}}$	14.9 (11.8–19.4)	22.8 (20.1–26.1)
$\;\sigma_{\text{female}}$	11.0 (10.4–11.8)	13.3 (12.5–14.1)
$\;\gamma_{\text{male}}$	0.47 (0.25–0.64)	–
$\;\gamma_{\text{female}}$	0.51 (0.44–0.58)	–
δ	50.1 (48.1–52.0)	50.0 (48.1–52.0)
Detection		
$\;\alpha_{0}$	−7.98 (−10.89 to −5.47)	−8.14 (−11.04 to −5.66)
$\;\alpha_{1}$	2.32 (1.65–3.10)	2.33 (1.67–3.12)
$\;\alpha_{2}$	0.52 (−1.10 to 2.10)	0.69 (−0.93 to 2.28)
$\;\sigma_{\det}$	5.11 (5.00–5.24)	5.10 (4.97–5.24)
Data augmentation		
$\;\psi_{\text{Male}}$	0.30 (0.19–0.47)	0.33 (0.21–0.51)
$\;\psi_{\text{Female}\;\mathrm{no}\;\text{offspring}}$	0.29 (0.16–0.48)	0.34 (0.19–0.56)
$\;\psi_{\text{Female with offspring}}$	0.23 (0.10–0.44)	0.27 (0.12–0.52)
Dependent offspring (1, 2, 3 offspring)		
$\;\omega_{1}$	0.29 (0.08–0.60)	0.28 (0.07–0.60)
$\;\omega_{2}$	0.61 (0.30–0.86)	0.61 (0.30–0.86)
$\;\omega_{3}$	0.07 (0.00–0.33)	0.07 (0.00–0.34)

*Note*: Data are from polar bear surveys during 25 March to 29 April 2015 in the eastern Chukchi Sea. Please refer to “*Methods*” section for detailed parameter descriptions.

**FIGURE 2:**
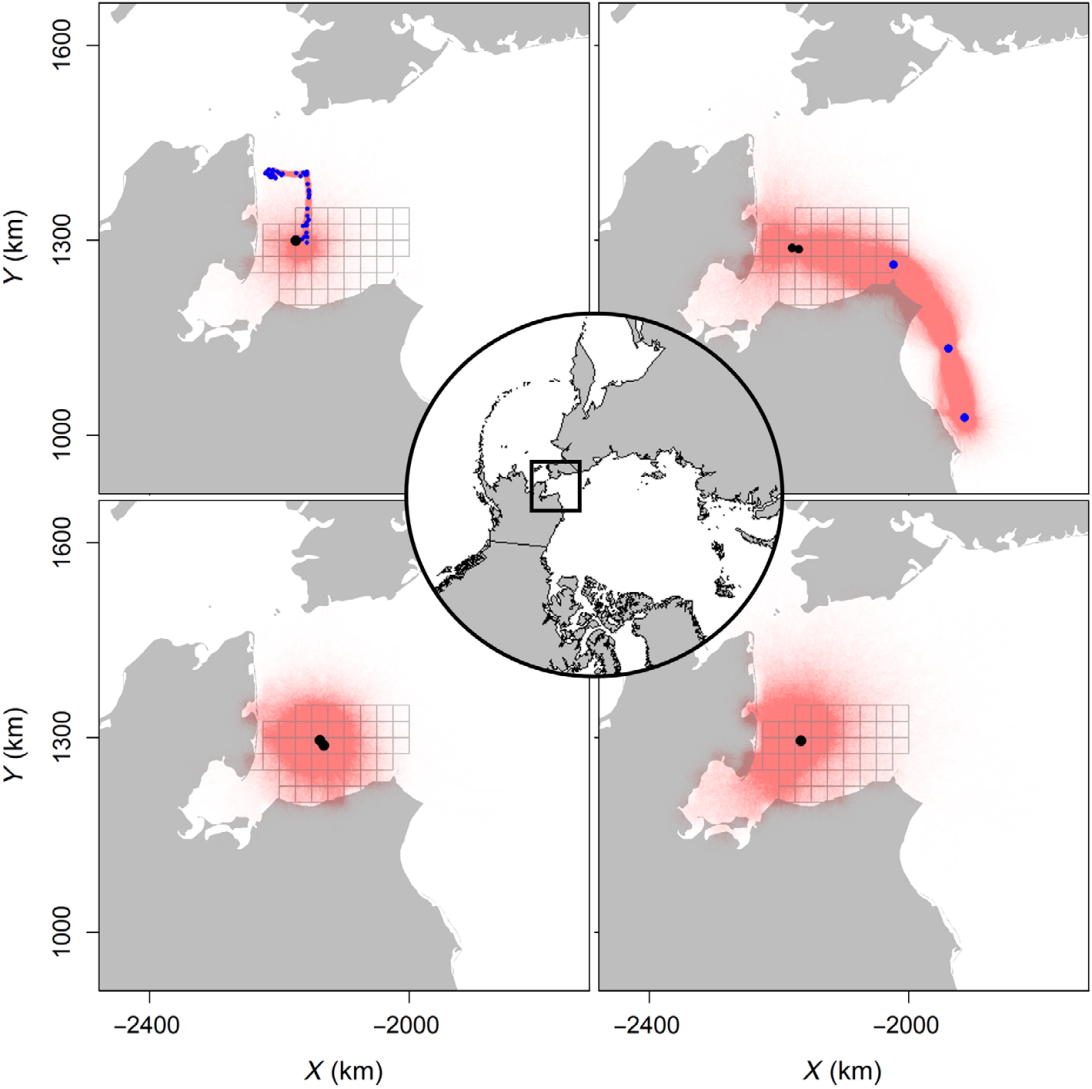
Posterior trajectories of four individual polar bears during the 36‐day sampling season (red; 5000 samples from posterior distributions). Female with GPS tag (top left), male with Argos tag (top right), female captured twice (bottom left), male captured once (bottom right). Capture and resight events (black dots), telemetry locations (blue dots), and surveyed area (25 × 25 km grid cells) are also shown.

Precision of posterior trajectories (i.e., the collection of an individual's estimated locations) varied by data quality and quantity for each individual and highlighted several advantages of the integrated SCR–movement model (Figure 2). First, the SCR–movement model allowed the estimation of individual‐level space use for all observed individuals (Figure 2). GPS collars resulted in precise posterior trajectories; however, location uncertainty prior to first capture remained noticeable (Figure 2). Posterior trajectories from bears with Argos tags displayed increased uncertainty due to fewer detections and less precise locations compared with GPS collars (Figure 2). Posterior trajectories for individuals without telemetry data displayed high levels of uncertainty due to the low number of recapture events, but locations were less likely to occur in areas with high survey effort (Figure 2). This point highlights an important aspect of SCR–movement models, in which all parameters, including latent locations, are informed by both detection and non‐detection data (e.g., telemetry, SCR detections, and SCR surveyed areas without detections).

**FIGURE 3:**
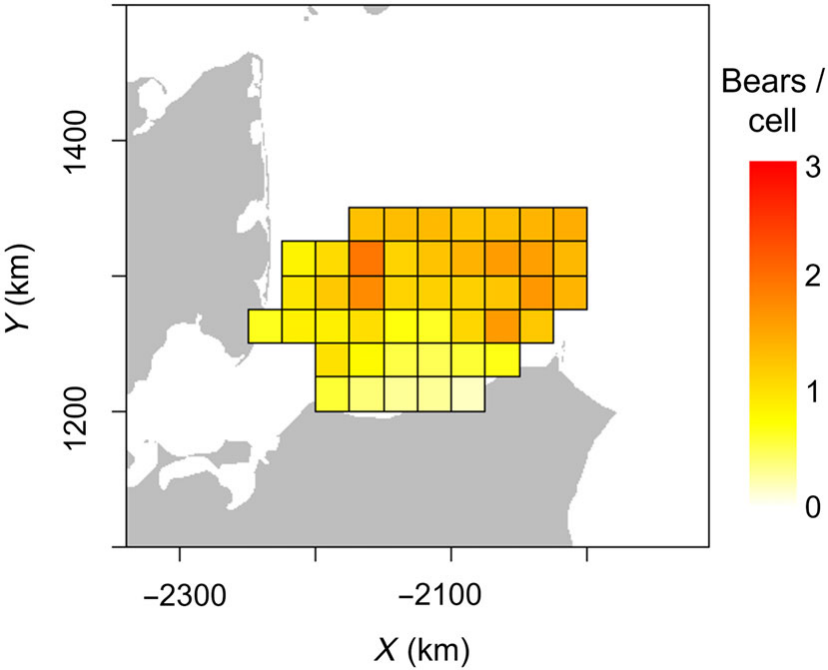
Mean daily polar bear abundance (independent bears + dependent offspring) per surveyed grid cell (25 × 25 km grid cells) during 25 March to 29 April 2015. Please refer to Figure 1 for data and survey area details.

The SCR–movement model estimated individual‐ and population‐level space use, including the effects of landscape characteristics on movement, abundance, and detection (Figures 2 and 3). For example, the movement process required that bears navigate the landscape on or near sea ice (Figure 2), replacing the standard SCR assumption of monotonically declining space use centered around a single activity center. Similarly, telemetry data informed the movement and detection processes, when non‐detections arose from individuals being outside the survey area and imperfect detection within the survey area (Figure 2).

We found that bear spatial densities varied from <0.75 bears/grid cell/day in nearshore cells and increased to 1.6–2.5 bears/grid cell/day for cells surrounded by sea ice (Figure 3). Variation in grid cell abundance reflected the observed data, when nearshore cells had fewer telemetry locations and fewer SCR detections relative to areas further offshore (Figures 1 and 3). Bear abundance outside the survey area converged toward an average of ≈1.14 independent bears per grid cell (95% CRI: 0.81–1.66) and ≈1.45 total bears per grid cell (95% CRI: 1.01–2.17).

**FIGURE 4:**
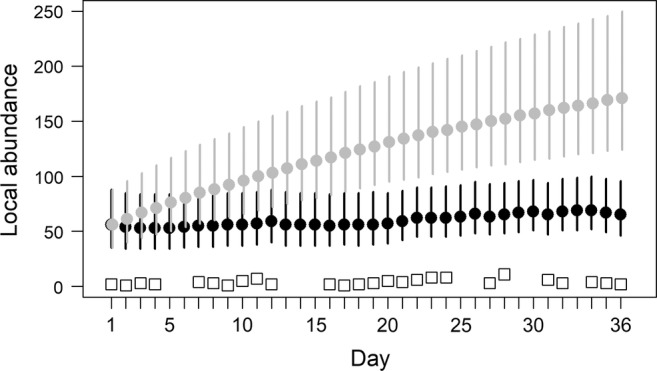
Daily (black dots) and cumulative (gray dots) polar bear abundance in the surveyed area (independent bears + dependent offspring) across the 36‐day sampling season (25 March to 29 April 2015). Values are posterior medians (95% credible intervals). The number of individuals observed on surveyed days are denoted by open squares.

Daily abundance estimates in the surveyed area ($N_{At}$) ranged from 53 bears (95% CRI: 34–84) to 69 bears (95% CRI: 52–100; Figure 4) and displayed no clear trend across days. The cumulative number of bears that used the surveyed area ($N_{At}^{*}$); however, increased through time (Figure 4). For example, the estimated cumulative number of bears that used the survey area by days 10, 20, and 36 were 96 (95% CRI: 66–145), 131 (95% CRI: 95–191), and 171 individuals (95% CRI: 124–250), respectively (Figure 4).

Estimated population structure of independent bears consisted of 0.54 males (95% CRI: 0.41–0.67), 0.30 females without dependent offspring (95% CRI: 0.19–0.43), and 0.16 females with dependent offspring (95% CRI: 0.08–0.27). The probabilities of one, two, or three dependent offspring were 0.29, 0.61, and 0.07, respectively, or an average of 1.72 dependent offspring per female with dependent offspring (Table 1). Detection probability increased with increasing survey effort ($\alpha_{1}$ = 2.32, 95% CRI: 1.65–3.10) and was higher during the first survey after capture (i.e., immediate trap response) but estimates of an immediate trap response overlapped zero ($\alpha_{2}$ = 0.52, 95% CRI: −1.10 to 2.10; Table 1).

### Simulation studies

The correlated random walk and bivariate normal random walk models performed well under sampling scenarios that included small numbers of captured individuals, low encounter rates, highly variable spatial survey effort, and habitat‐influenced movement processes (Appendix S4: Table S1). Percentage relative bias was ≤0.2% for movement parameters (σ, γ) and ≤4% for detection parameters ($\alpha_{0}$, $\alpha_{1}$, $\sigma_{\det}$; Appendix S4: Table S1). For correlated random walk simulations, daily true, and estimated abundances within the survey area averaged 54.2 and 55.5 individuals, respectively, with mean daily abundance estimates within ±2 individuals of the true value (Appendix S4: Table S1). Daily local abundance estimates in the bivariate normal random walk simulation were even closer to true values (±1 individual; Appendix S4: Table S1). For both simulations, credible interval coverage ranged from 0.93 to 1.00 for parameters and 0.92 to 1.00 for derived abundances (Appendix S4: Table S1).

## DISCUSSION

We describe an integrated SCR–movement model to connect individual‐level movement dynamics to population‐level spatiotemporal abundances. Our polar bear case study and simulation examples illustrate advantages of incorporating greater movement realism into SCR models, and the ability of SCR–movement models to investigate shared hypotheses across movement, population, and landscape ecology (McClintock et al., 2021). Large movements of individual polar bears in our case study demonstrated the importance of SCR–movement models, which revealed sex‐specific movement processes, highly dynamic detection probabilities, and spatial variation in bear density as a function of landscape characteristics. Together, integrated SCR–movement models provide new opportunities to explore ecological questions and address persistent study design challenges.

Our estimates of polar bear density in the eastern Chukchi Sea (≈0.002 bears/km^2^; 95% CRI: ≈0.001–0.004) align with average estimates for the period 2008–2016 from an integrated population model encompassing a similar region (0.0030 bears/km^2^, 95% CRI: 0.0016–0.0060; Regehr et al., 2018). Many polar bear abundance studies utilize capture–recapture methods (Bromaghin et al., 2015; Hamilton & Derocher, 2019; Lunn et al., 2016; Regehr et al., 2018), although comparisons among non‐spatial abundance estimates are difficult because the effective study population is an unknown function of spatial coverage, survey duration, and movement (Kendall et al., 1997; Lunn et al., 2016). This can lead to substantive bias in estimates of demographic parameters used for management (Regehr et al., 2009). Our SCR–movement model solves this challenge by linking the movement and abundance processes to directly estimate density within a prescribed spatial region and temporal period. For example, we estimated abundance in the survey area of ≈53–69 bears/day, whereas the cumulative number of bears exposed to sampling (i.e., “effective study population”) increased daily due to bears moving into and out of the survey area (Figure 4). Although our case study focused on a polar bear abundance and movement in nearshore area, approaches integrating aerial surveys and telemetry data provide new opportunities to link movement ecology to spatial planning and conservation efforts including marine protected areas (e.g., Conn et al., 2021; Lennox et al., 2019; Ogburn et al., 2017) and offshore energy development that increasing affect marine megafauna (Sequeira et al., 2019; Wilson et al., 2014).

A unified modeling framework for movement and population ecology from an individual‐based movement perspective has multiple advantages (Nathan et al., 2008). Specifically, it facilitates exploration into the causes and patterns of movement, with far reaching consequences on emergent properties such as spatiotemporal abundance, species interactions, and population‐level responses to management actions (McClintock et al., 2021; Morales et al., 2010). For example, SCR–movement models provided more realistic space use and observation processes in our polar bear case study, whereby detections arose from the combination of individuals and observers making directed movements through the landscape, instead of bears randomly moving around an activity center and observers remaining in fixed locations (Royle et al., 2013). Furthermore, individual‐level locations ($s_{\mathrm{it}}$) are directly linked to spatiotemporal abundances ($N_{At}$). As such, individual‐level movement and population‐level abundance are linked, where processes formulated at one level correspond to properties exhibited at the other (Hooten et al., 2017; McClintock et al., 2021; Royle et al., 2013; Turchin, 1998).

Studies on the location and spatial distribution of individuals use a variety of sampling methods. A dichotomy in conceptual approaches often exists, however, in which studies focus on quantifying either the movements of individual organisms (i.e., Lagrangian perspective) or the number of animals that use or move through a fixed location (i.e., Eulerian perspective; Turchin, 1998). SCR–movement models bridge these conceptual approaches to unify individual‐ and population‐level processes. Here, the shared point process model allows quantification of both individual movement (e.g., step lengths, persistence, velocity) and changes in densities or numbers of individuals at defined spatial locations (e.g., the state space or region within the state space). Quantifying spatiotemporal landscape use and spatial distributions of populations (which is a result of individual‐level movement) is crucial to understanding the responses to ecosystem change. Potential applications of SCR–movement model concepts abound, with logical extensions focused on incorporating habitat‐driven movement and resource selection (e.g., step selection functions; Avgar et al., 2016), integration of auxiliary data at various spatial scales, and multiyear open‐population SCR models to explore the effects of within‐ and among‐year movement on population dynamics (Bischof et al., 2020; Glennie et al., 2019). The unification of movement and population ecology also has numerous applied benefits including an improved understanding of how and why populations respond to management actions related to marine spatial planning (e.g., Lennox et al., 2019; Ogburn et al., 2017), energy development (e.g., Wilson et al., 2014), and conservation of migratory species (e.g., Sequeira et al., 2019).

Spatial processes are an important component of ecological predictions, but quantifying relationships between individual‐level movement and changes in abundance is difficult in conventional demographic and movement models (Hooten et al., 2017; Morales et al., 2010; Royle et al., 2013). SCR–movement models explicitly describe the mechanistic links between movement and population dynamics, providing new ways to investigate how spatiotemporal patterns of abundance are shaped by individual‐level movement. For example, it is generally accepted that movement dynamics respond to changing resource availability and density‐dependent factors, which affect individual‐level fitness and ultimately population dynamics (Ims & Andreassen, 2005; Nathan et al., 2008). SCR–movement models formalize these topics in a unified approach to explicitly investigate connections between movement and population dynamics, providing a substantial progression toward connecting movement, landscape, and population ecology (McClintock et al., 2021).

Our goal in this paper was to systematically describe and demonstrate the building blocks of SCR–movement models, where parameters summarize movement patterns, individual‐level space use, and spatiotemporal densities. SCR–movement models offer an approach to quantify the causes, patterns, and consequences of animal movement, abundance, and population dynamics that are central to understanding and managing populations and the landscapes that support them (Kays et al., 2015; Morales et al., 2010; Nathan et al., 2008). Future applications of SCR–movement models that incorporate a variety of spatial data collection protocols will increase their applicability and the ability to connect movement and population dynamics.

## CONFLICT OF INTEREST

The authors declare no conflict of interest.

## Supporting information


Appendix S1

Appendix S2

Appendix S3

Appendix S4

Data S1

Data S2

## Data Availability

Data (Regehr et al., 2022a) is available in Dryad at https://doi.org/10.5061/dryad.pk0p2ngq7. Code (Regehr et al., 2022b) is available in Zenodo at https://doi.org/10.5281/zenodo.6505053.
