# Supplementary material for: Modeling spatiotemporal abundance and movement dynamics using an integrated spatial capture–recapture movement model
Source: Ecology. 2022 Jul 15;103(10):e3772. doi: 10.1002/ecy.3772 (PMC9787655; doi:10.1002/ecy.3772)
Supplement: Supplementary file 1 — Appendix S1 [file ECY-103-e3772-s001.pdf]

APPENDIX S1

Hostetter, N.J., Regehr, E.V., Wilson, R.R., Royle, A.J., Converse, S.J., Modeling spatiotemporal abundance and movement dynamics using an integrated spatial capture-recapture movement model. *Ecology*

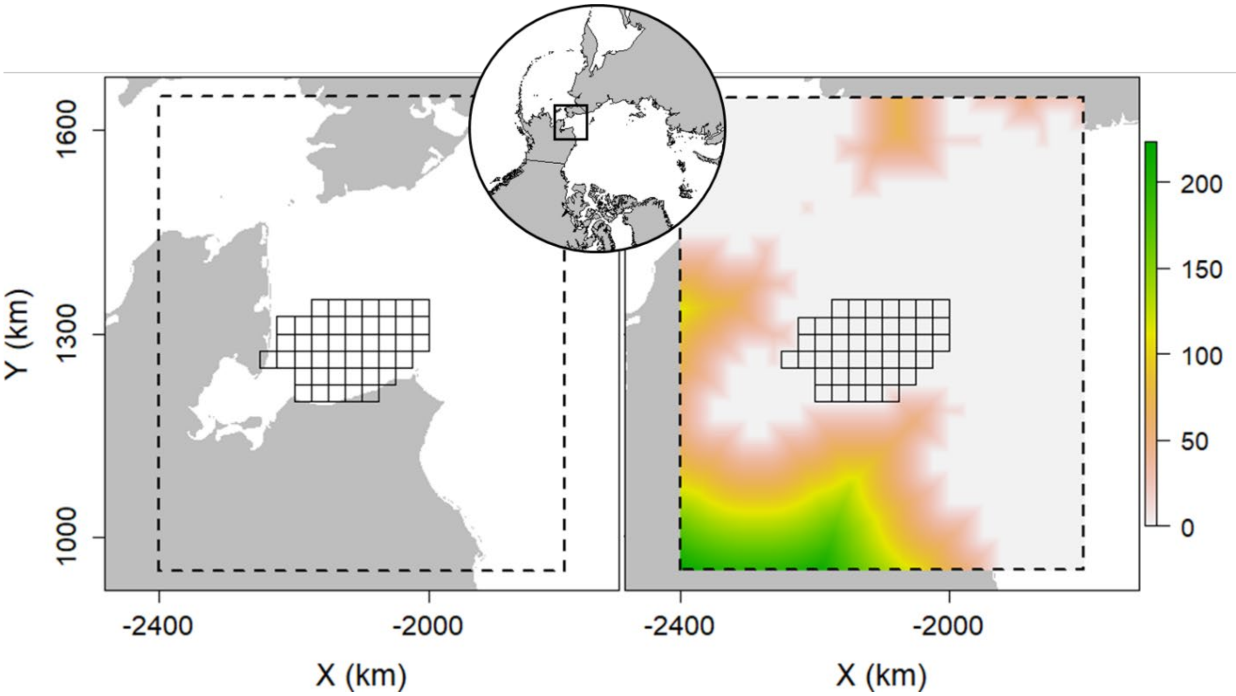

Figure S1. Polar bear survey area (25×25 km grid cells), state-space (dashed polygon), and covariate describing distance to edge of sea ice (km; right).
