# Supplementary material for: Modeling spatiotemporal abundance and movement dynamics using an integrated spatial capture–recapture movement model
Source: Ecology. 2022 Jul 15;103(10):e3772. doi: 10.1002/ecy.3772 (PMC9787655; doi:10.1002/ecy.3772)
Supplement: Supplementary file 2 — Appendix S2 [file ECY-103-e3772-s004.pdf]

APPENDIX S2

Hostetter, N.J., Regehr, E.V., Wilson, R.R., Royle, A.J., Converse, S.J., Modeling spatiotemporal abundance and movement dynamics using an integrated spatial capture-recapture movement model. *Ecology*

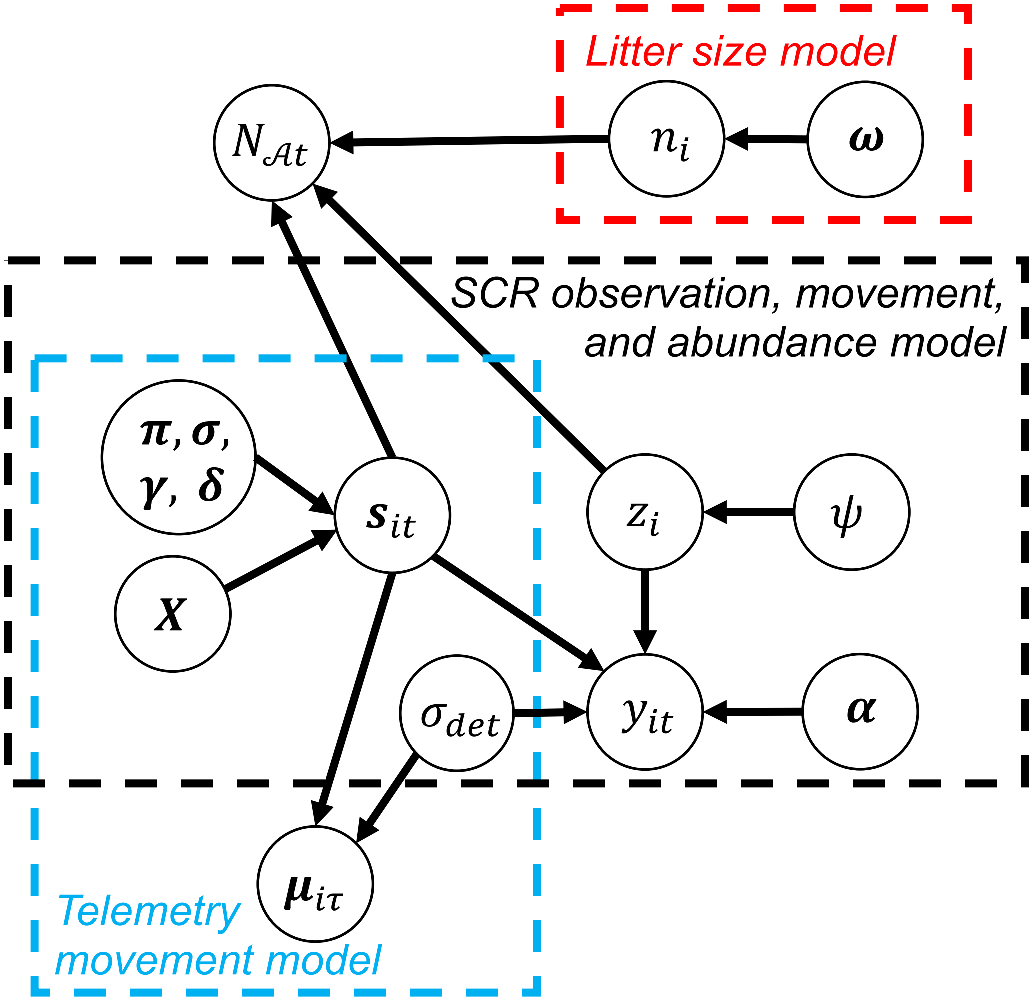

Figure S1. Directed acyclic graph (DAG) of the integrated SCR-movement model using spatial capture-recapture data (y_{it}), telemetry location data ($\boldsymbol{\mu}_{i\tau}$), landscape covariates ($\boldsymbol{X}$, e.g., sea ice), and counts of dependent cubs (n_i). SCR data (y_{it}) directly link to detection parameters ($\boldsymbol{\alpha}$), daily average locations ($\boldsymbol{s}_{it}$), space use around daily average locations (σ_{det}), and data augmentation indicator variables (z_i). Both SCR and telemetry data inform movement parameters ($\boldsymbol{\pi}$, $\boldsymbol{\sigma}, \boldsymbol{\gamma}$, δ)

that describe how average locations change through time, which can be a function of landscape- or individual-level covariates. Counts of cubs per female inform litter size parameters ($\boldsymbol{\omega}$). Daily abundance in the surveyed area ($N_{\mathcal{A}t}$) is a derived metric but retained in the DAG for clarity, and represents the number of independent bears and dependent cubs in the study area on day t. For simplicity, some parameters are combined into shared circles. This DAG describes the Correlated Random Walk model described in the text when $\boldsymbol{\gamma} > 0$ and the Random Walk model when $\boldsymbol{\gamma} = 0$, with numerous generalizations and extensions possible. See Methods for complete description of data, model, and parameters.
