# Supplementary material for: Modeling spatiotemporal abundance and movement dynamics using an integrated spatial capture–recapture movement model
Source: Ecology. 2022 Jul 15;103(10):e3772. doi: 10.1002/ecy.3772 (PMC9787655; doi:10.1002/ecy.3772)
Supplement: Supplementary file 3 — Appendix S3 [file ECY-103-e3772-s006.pdf]

APPENDIX S3

Hostetter, N.J., Regehr, E.V., Wilson, R.R., Royle, A.J., Converse, S.J., Modeling spatiotemporal abundance and movement dynamics using an integrated spatial capture-recapture movement model. *Ecology*

Table S1. Comparison of parameter estimates (median, 95% credbile interval) for movement, detection, and abundance within the surveyed area ($N_{\mathcal{A}}$) from an integrated SCR-movement model using a correlated random walk and a traditional SCR model that does not integrate a movement process. Data are from polar bear surveys during 25 March – 29 April 2015 in the eastern Chukchi Sea. See Methods for detailed data and parameter descriptions.

Parameter	SCR-movement	SCR
Between occasion movement		
σ_{male}	14.9 (11.8 – 19.4)	–
σ_{female}	11.0 (10.4 – 11.8)	–
γ_{male}	0.47 (0.25 – 0.64)	–
γ_{female}	0.51 (0.44 – 0.58)	–
δ	50.1 (48.1 – 52.0)	–
Detection		
α_0	-7.98 (-10.89 – -5.47)	-10.87 (-12.78 – -9.23)
α_1	2.32 (1.65 – 3.10)	1.57 (1.14 – 2.70)
α_2	0.52 (-1.10 – 2.10)	1.76 (0.69 – 2.70)
σ_{det}	5.11 (5.00 – 5.24) †	45.14 (44.08 – 46.20) †
Abundance in study area		
$N_{\mathcal{A}t}$ (daily)	53 (34 – 84) ‡	–
$N^*_{\mathcal{A}T}$ (cumulative)	171 (124 – 250) ‡	–
$N^{SCR}_{\mathcal{A}}$	–	114 (85 – 182) §

† Definitions of σ_{det} vary between modelling approaches (Royle et al. 2016, McClintock et al. 2021). In the C-RW model, σ_{det} describes random noise around a daily location (i.e., space use around a daily location). In the SCR model, σ_{det} describes the space use around an individual's single activity center for the entire study period. Thus, σ_{det} captures *daily* random noise in the C-RW model and *seasonal* bivariate normal space use in the SCR model.

‡ $N_{\mathcal{A}t}$ (daily) and $N^*_{\mathcal{A}T}$ (cumulative) are the daily and cumulative polar bear abundance in the survey area. Daily abundance varies by day, with an average of 53 bears per day (see Figure 4).

§ $N_{\mathcal{A}}^{SCR}$ is the number of activity centers within the surveyed area ($\mathcal{A}$) estimated from a standard SCR model. In the standard SCR model, individuals have a single (latent) activity center that reflects their average location during the study. As such, an individual may use the surveyed area but have an average location outside the survey area, and are therefore not be included in $N_{\mathcal{A}}^{SCR}$ (see Figure 1 for examples of telemetered individuals that spend the majority of time outside the surveyed area). As expected, the estimated number of activity centers in the surveyed area from the standard SCR model ($N_{\mathcal{A}}^{SCR}$) is between estimates of daily abundance ($N_{\mathcal{A}t}$) and cumulative abundance ($N^*_{\mathcal{A}T}$) in the SCR-movement model (see also Royle et al. 2016).

LITERATURE CITED

McClintock, B. T., B. Abrahms, R. B. Chandler, P. B. Conn, S. J. Converse, B. Gardner, N. J. Hostetter, and D. S. Johnson. 2021. An integrated path for spatial capture-recapture and animal movement modeling. Ecology.

Royle, J. A., A. K. Fuller, and C. Sutherland. 2016. Spatial capture–recapture models allowing

Markovian transience or dispersal. Population Ecology 58:53–62.
