# Supplementary material for: Modeling spatiotemporal abundance and movement dynamics using an integrated spatial capture–recapture movement model
Source: Ecology. 2022 Jul 15;103(10):e3772. doi: 10.1002/ecy.3772 (PMC9787655; doi:10.1002/ecy.3772)
Supplement: Supplementary file 4 — Appendix S4 [file ECY-103-e3772-s002.pdf]

APPENDIX S4

Hostetter, N.J., Regehr, E.V., Wilson, R.R., Royle, A.J., Converse, S.J., Modeling spatiotemporal abundance and movement dynamics using an integrated spatial capture-recapture movement model. *Ecology*

Table S1. Detailed simulation results comparing data generating values (True), expected values (Mean), percent relative bias (% Relbias), and 95% credible interval coverage (Cov.) from 100 simulated data sets generated using a correlated random walk or bivariate normal randow walk movement process. Realized survey area abundance on day *t* (N[t]) and the cumulative number of polar bears that used the survey area (N_T^*) are derived statistics influenced by movement and the total number of individuals in the state-space (N), as such True values are averages across all simulations. See Methods for detailed parameter definitions.

	Correlated Random Walk				Bivariate Normal Random Walk			
Parameter	True	Mean	% Relbias	Cov.	True	Mean	% Relbias	Cov.
Movement								
σ	15.00	15.00	-0.01	0.95	15.00	14.97	-0.22	0.93
γ	0.50	0.50	-0.92	0.96	0.00	–	–	–
δ	50.00	50.01	0.02	1.00	50.00	50.01	0.03	1.00
Detection								
α_0	-8.00	-8.16	1.96	0.95	-8.00	-8.33	4.11	0.94
α_1	2.50	2.53	1.16	0.95	2.50	2.58	3.02	0.93
σ_{det}	5.00	5.00	0.09	0.93	5.00	5.00	-0.06	0.94
Abundance								
N	500	525.8	5.15	0.94	500	504.1	0.81	0.96
Daily local abundance								
N[1]	55.7	56.8	2.80	0.96	54.8	54.3	-0.52	0.96
N[2]	52.8	54.6	4.11	0.96	51.8	52.1	1.03	0.96

N[3]	52.5	54.2	3.97	0.96	52.7	52.6	0.23	0.96
N[4]	52.7	54.4	3.78	0.96	52.5	52.8	1.16	0.95
N[5]	53.1	54.6	3.13	0.98	52.3	52.9	1.85	0.94
N[6]	53.2	54.6	2.78	0.99	52.6	53.0	1.24	0.96
N[7]	53.0	54.7	3.93	0.94	53.0	53.1	0.68	0.96
N[8]	53.0	55.0	4.49	0.92	53.2	53.3	0.42	0.96
N[9]	53.6	55.2	3.65	0.94	53.5	53.3	-0.02	0.94
N[10]	54.0	55.3	3.07	0.95	54.3	53.6	-1.18	0.94
N[11]	53.8	55.3	3.34	0.96	53.6	53.5	-0.14	0.97
N[12]	54.3	55.5	2.49	0.95	53.5	53.5	0.35	0.97
N[13]	54.4	55.5	2.32	0.97	53.5	53.7	0.90	0.94
N[14]	54.8	55.6	2.06	0.95	54.0	53.7	-0.38	0.95
N[15]	54.5	55.5	2.47	0.97	54.3	53.8	-0.53	0.96
N[16]	54.8	55.8	2.32	0.97	54.5	53.9	-0.77	0.92
N[17]	54.3	55.9	3.33	0.95	54.2	53.7	-0.53	0.94
N[18]	54.7	55.9	2.71	0.95	54.0	53.9	0.19	0.94
N[19]	55.6	56.2	1.42	0.98	53.7	53.9	0.67	0.98
N[20]	54.8	56.0	2.50	0.99	53.8	53.7	0.12	0.98
N[21]	55.2	56.1	2.13	1.00	54.1	53.9	-0.09	0.94
N[22]	55.3	56.2	2.19	0.98	53.7	54.1	1.33	0.92
N[23]	54.8	56.3	2.90	0.96	54.0	54.2	0.91	0.95
N[24]	55.0	56.2	2.39	0.99	54.1	54.4	1.14	0.95
N[25]	54.9	56.3	2.88	0.96	54.0	54.5	1.28	0.95
N_T^*	143.0	147.1	2.84	0.96	105.9	105.4	-0.60	0.95
