# Supplementary material for: Modeling spatiotemporal abundance and movement dynamics using an integrated spatial capture–recapture movement model
Source: Ecology. 2022 Jul 15;103(10):e3772. doi: 10.1002/ecy.3772 (PMC9787655; doi:10.1002/ecy.3772)
Supplement: Supplementary file 5 — Data S1 [file ECY-103-e3772-s003.zip › MetadataS1.pdf]

Hostetter, N.J., Regehr, E.V., Wilson, R.R., Royle, A.J., Converse, S.J. 2022. Modeling spatiotemporal abundance and movement dynamics using an integrated spatial capture–recapture movement model. *Ecology.*

Data S1

R scripts and shapefiles to simulate data and fit the integrated spatial capture-recapture (SCR) movement model.

Authors

Nathan J. Hostetter
Washington Cooperative Fish and Wildlife Research Unit, School of Aquatic and Fishery Sciences, University of Washington, Seattle, WA, USA. *Current address*: U.S. Geological Survey, North Carolina Cooperative Fish and Wildlife Research Unit, Department of Applied Ecology, North Carolina State University, Raleigh, NC, USA
njhostet@ncsu.edu

Eric V. Regehr
Applied Physics Laboratory, Polar Science Center, University of Washington, Seattle, WA, USA

Ryan R. Wilson
Marine Mammals Management, United States Fish and Wildlife Service, Anchorage, AK, USA

J. Andrew Royle
U.S. Geological Survey, Eastern Ecological Science Center, Laurel, MD, USA

Sarah J. Converse
U.S. Geological Survey, Washington Cooperative Fish and Wildlife Research Unit, School of Environmental and Forest Sciences & School of Aquatic and Fishery Sciences, University of Washington, Seattle, WA, USA

File list (files found within DataS1.zip)

```
sim_SCR_mvmt.R
sim_SCR_mvmt_SamplersAndFunctions.R
effortLines_raw_final_06july16.shp
```

gridCells_final_06july16.shp

Description

sim_SCR_mvmt.R - This R script simulates SCR-movement data described in the manuscript, including initial abundance, initial distribution, random walk movement processes, telemetry, and SCR detection processes.

sim_SCR_mvmt_SamplersAndFunctions.R - This R script provides the MCMC functions for the SCR-movement model described in sim_SCR_mvmt.R. While nearly all aspects can be expressed in common BUGS language, these functions greatly improve MCMC efficiency.

effortLines_raw_final_06july16.shp - Helicopter track files used to quantify search effort.

gridCells_final_06july16.shp - Grid cells used to discretize the state-space
