# Supplementary material for: Modeling spatiotemporal abundance and movement dynamics using an integrated spatial capture–recapture movement model
Source: Ecology. 2022 Jul 15;103(10):e3772. doi: 10.1002/ecy.3772 (PMC9787655; doi:10.1002/ecy.3772)
Supplement: Supplementary file 6 — Data S2 [file ECY-103-e3772-s005.zip › MetadataS2.pdf]

Hostetter, N.J., Regehr, E.V., Wilson, R.R., Royle, A.J., Converse, S.J. 2022. Modeling spatiotemporal abundance and movement dynamics using an integrated spatial capture-recapture movement model. *Ecology*.

Data S2

R scripts and data files to fit the case study integrated spatial capture-recapture (SCR) movement model.

Authors

Nathan J. Hostetter
Washington Cooperative Fish and Wildlife Research Unit, School of Aquatic and Fishery Sciences, University of Washington, Seattle, WA, USA. *Current address*: U.S. Geological Survey, North Carolina Cooperative Fish and Wildlife Research Unit, Department of Applied Ecology, North Carolina State University, Raleigh, NC, USA
njhostet@ncsu.edu

Eric V. Regehr
Applied Physics Laboratory, Polar Science Center, University of Washington, Seattle, WA, USA

Ryan R. Wilson
Marine Mammals Management, United States Fish and Wildlife Service, Anchorage, AK, USA

J. Andrew Royle
U.S. Geological Survey, Eastern Ecological Science Center, Laurel, MD, USA

Sarah J. Converse
U.S. Geological Survey, Washington Cooperative Fish and Wildlife Research Unit, School of Environmental and Forest Sciences & School of Aquatic and Fishery Sciences, University of Washington, Seattle, WA, USA

File list (files found within DataS2.zip)

```
CaseStudyData.txt
SCR_mvmt_CaseStudy.R
SCR_mvmt_CaseStudy_SamplersAndFunctions.R
```

Description

`CaseStudyData.txt` - This text file contains the data associated described in the cast study.

`SCR_mvmt_CaseStudy.R` - This R script loads and analyzes SCR-movement data described in the cast study, including initial abundance, initial distribution, random walk movement processes, telemetry, and SCR detection processes.

`SCR_mvmt_CaseStudy_SamplersAndFunctions.R` - This R script provides the MCMC functions for the SCR-movement model described in SCR_mvmt_CaseStudy.R. While nearly all aspects can be expressed in common BUGS language, these functions greatly improve MCMC efficiency.
